# Soil Potassium Application Ameliorates Drought-Induced Seed Yield Loss and Enhances Nutritional and Seed Oil Quality in Sesame (*Sesamum indicum* L.)

**DOI:** 10.3390/plants15121830

**Published:** 2026-06-12

**Authors:** Zehua Wan, Yiming Xu, Sheng Fang

**Affiliations:** 1 Key Laboratory of Crop Physiology, Ecology, and Genetic Breeding, Ministry of Education/College of Agronomy, Jiangxi Agricultural University, Nanchang 330045, China; 15734711673@stu.jxau.edu.cn (Z.W.); yimingxu@stu.jxau.edu.cn (Y.X.); 2 Jingan County Agriculture and Rural Affairs Bureau, Yichun 330699, China

**Keywords:** sesame, potassium fertilization, water deficit, oil yield, seed nutritional quality

## Abstract

Sesame is a considerable oilseed crop, but its growth and production are restricted by drought. Potassium (K) is well known for its mitigating effects against drought. Here, two consecutive years of experiments were conducted with varying K fertilizer rates (0, 60, and 120 kg K_2_O ha^−1^) under well-watered and drought conditions to evaluate the impacts of K on sesame seed quality. The results demonstrated that, compared to well-watered conditions, drought caused a decline in seed oil content (5.9–8.6%) but inversely induced an increase in seed K (8.5–23.8%), lignans (10.2–21.6%), and essential amino acids over a period of 2 years. Potassic fertilizer significantly increased seed K, oil, and lignans contents, aligning with ameliorative oil and protein yield relative to K deficiency plants under drought. Moreover, K supply (especially 120 kg K_2_O ha^−1^) increased proline and tryptophan contents by 5.2% and 4.9% under drought compared to the plants without K application, which contributed to producing lignans and enhancing the capacity against oxidative changes. Under drought, 60 and 120 kg K_2_O ha^−1^ application significantly increased linoleic (5.5–9.3%), and stearic acids (7.1–13.7%) content while decreasing palmitic (5.3–14.7%), oleic (4.6–6.4%), and linolenic acids (4.8–11.9%) content, respectively, thereby increasing the ratio of unsaturated to saturated fatty acids and unsaturation index compared with control without K. Overall, K application at the rate of 120 kg K_2_O ha^−1^ could be considered as a practical and straightforward strategy to improve the quality of sesame seed products by increasing seed K, oil, lignans, linoleic acid, and unsaturated index for pharmaceutical and food purposes in areas encountering drought stress.

## 1. Introduction

Sesame (*Sesamum indicum* L.) seed contains considerable amounts of oil, protein and secondary metabolites such lignans, flavonoid, and phenolic compounds [1]. It is widely used in food, animal feed, pharmaceuticals and cosmetics, and occupies a prominent position in edible oil industries [2]. Sesame oil contains a high proportion of unsaturated fatty acids (UFAs, 80%), linoleic and oleic acids, and a low level of α-linolenic acid. It also has indispensable health-promoting effects such as reducing cholesterol and blood pressure levels, providing neuroprotective function against hypoxia and brain damage, and reducing the likelihood of developing certain types of cancer [3,4]. The predominant fatty acid present with sesame seeds is linoleic acid (C18:2), followed by oleic acid (C18:1), palmitic acid (C16:0), and stearic acid (C18:0) [5]. Additionally, sesame oil is more resilient to oxidative changes than other vegetable oils because of its sesamin, sesamolin and α-tocopherol ingredients [6]. Sesame seeds serve as an important source of essential fatty acids, including linoleic acid, which must be obtained from the diet, as it cannot be synthesized endogenously [7]. Therefore, the industrial and economic values of sesame are very high, and it is very popular among many people.

In oilseed crops such as sesame, drought stress further disrupts the synthesis and accumulation of storage lipids and proteins, and alters the composition of fatty acids and secondary metabolites, ultimately leading to reduced seed yield and compromised quality [8,9]. The most pronounced effect of drought stress is growth retardation as well as yield and quality losses [10,11,12]. Drought stress during the reproductive growth stage is particularly devastating. It severely impairs reproductive development, leading to substantial yield losses [13] and compromised seed quality [14]. Drought stress will reduce plant pollination, inhibit grain filling, and change grain quality characteristics [9,15] by decreasing oil and linoleic acid content [14]. Plants exhibit a series of complex physiological and biochemical responses to cope with drought stress, including the accumulation of osmo-protectants (e.g., proline, soluble sugars) for osmotic adjustment, activation of antioxidant defense systems to scavenge reactive oxygen species (ROS) and mitigate oxidative damage [16], and adjustments in photosynthetic carbon assimilation and nutrient metabolism to maintain basic growth [13]. In addition, drought stress reduced seed sesamin content and increased sesamolin in most of the genotypes investigated [6]. The seed lignans (sesamin and sesamolin) content depends on differences in genetic background and environmental factors, particularly under drought stress conditions [17]. Implementing economically viable strategies to maintain sesame yield and quality under drought conditions is essential for sesame production in arid regions.

Various strategies have been developed to enhance plant drought tolerance, including genetic breeding and genetic engineering for drought-tolerant cultivars [18], application of plant growth regulators and osmo-protectants [19], soil amendment with biochar, and organic fertilizers [11], and optimized nutrient management [20]. Among these, potassium (K) application is recognized as an economical, straightforward and effective agronomic strategy to ameliorate drought stress in crops, as K is involved in regulating multiple physiological and biochemical processes related to stress resistance [21]. To comprehensively realize sesame’s production and quality potential in seed, the soil must provide adequate amounts of nutrients. K performs a vital role in various physio-biochemical processes, such as reducing oxidative damage, promoting sucrose metabolism, and thereby enhancing drought tolerance [22,23]. K deficiency alters the distribution of nitrogen compounds between free amino acids (FAAs) and proteins [24], and concurrently changes oil properties. Lipids are synthesized in various intracellular organelles using carbon skeletons derived from sugars provided by leaves, whereas seed storage proteins are synthesized using FAA imported directly from the source organs. K also plays imperative roles in fatty acids and lipids metabolism, since K could improve oleic, lauric, palmitic, and linolenic acids [25]. Therefore, maintaining adequate potassium nutrition in plants is crucial for acclimatizing to drought [26]. The advantages of K application have been observed in other crops such as rape [27], cotton [28], and sunflower [20]. Similarly, K also improves the seed oil and protein yields in sesame under drought conditions [22].

Overall, evidence for the beneficial role of potassium (K) in improving crop drought resistance is promising [19,29,30]. It is an overriding consideration to improve sesame seed traits with high seed yield and oil content in those limited availability for irrigated production areas. Although previous studies confirmed that K improves drought tolerance and seed yield in sesame, few studies have explored the regulatory effects and internal mechanisms of K on fatty acid composition, lignans, amino acids, and their intrinsic relationships under drought. Can exogenous potassium application alleviate the negative impacts of drought on sesame seed quality? The primary objective of this experiment was to explore the effects of different potassium application rates on seed yield, oil, protein, lignans, amino acids and fatty acid profiles of sesame under well-watered and drought stress conditions, and to screen out the optimal K fertilizer rate suitable for sesame cultivation in drought-prone areas. We hypothesized that (1) exogenous potassium application can alleviate the adverse effects of drought stress on sesame seed quality by regulating the accumulation of seed potassium, and the alleviation effect is positively correlated with the potassium application rate within a certain range; (2) potassium fertilizer can regulate the fatty acid composition of sesame seeds under drought stress by enhancing the activity of fatty acid desaturase, thereby increasing the content of unsaturated fatty acids and improving the nutritional quality of sesame oil. To test this hypothesis, a two-year pot experiment was conducted to investigate the effects of different K levels to clarify the effects of soil-applied K on seed yield, oil, protein, lignans, amino acids and fatty acid profiles of sesame under well-watered and drought conditions, and to screen the optimal K application rate for sesame production in drought-prone regions.

## 2. Results

### 2.1. Changes in Seed K Content

**Figure 1:**
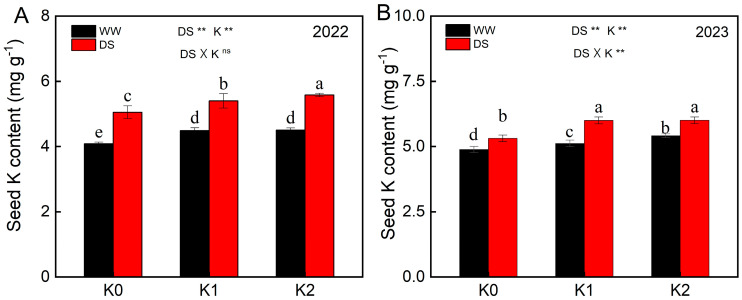
Effects of K application on seed K content in 2022 (**A**) and 2023 (**B**) under different water treatments. K0, K1, and K2 were designated for 0, 60, and 120 kg K_2_O ha^−1^, respectively. WW—well-watered; DS—drought stress. Data are expressed as the means of three replications (mean ± SE, n = 3). Different lowercase letters show significant differences at *p* < 0.05 level. ** indicates significant difference at *p* < 0.01 probability level, respectively. ns means not significant.

Two-way ANOVA showed that drought stress (DS), K fertilizer rate, and their interaction (DS × K) all had significant effects on seed K content (*p* < 0.05), though the interaction effect was only significant in 2023 (*p* < 0.01). The two-year results showed that drought stress (DS) induced a considerable increase (*p* < 0.05) in seed K content compared to well-watered (WW) plants, with increments of 8.5–23.7%, 17.3–20.2%, and 11.0–23.8% in K0, K1, and K2 treatments, respectively (Figure 1A,B). Concurrently, the main effect of K was also significant, with seed K content increasing in a dose-dependent manner under both WW and DS conditions (*p* < 0.05). Under drought stress, seed K content was increased by 6.9–13.0% and 10.4–13.1% for K1 and K2 treatments compared with K0 over a period of two years.

### 2.2. Changes in Oil and Protein Content

**Figure 2:**
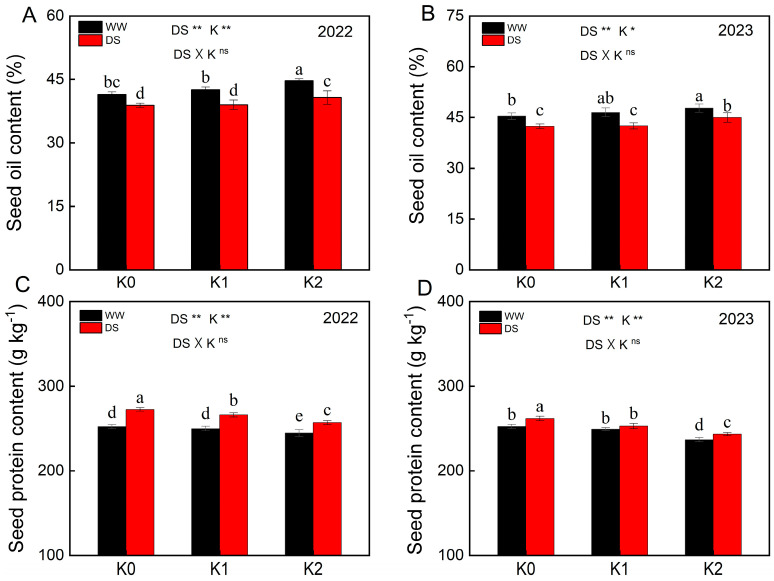
Effects of K application on seed oil (**A**,**B**) and protein (**C**,**D**) content under different water treatments. K0, K1, and K2 were designated for 0, 60, and 120 kg K_2_O ha^−1^, respectively. WW—well-watered; DS—drought stress. Data are expressed as the means of three replications (mean ± SE, n = 3). Different lowercase letters show significant differences at *p* < 0.05 level. * and ** indicates significant difference at *p* < 0.05 and *p* < 0.01 probability level, respectively. ns means not significant.

Drought stress significantly reduced seed oil content but increased protein content (*p* < 0.05), while K application mitigated the reduction in oil content (Figure 2A,B). Over two years, compared to WW treatment, DS induced reductions in seed oil content by 6.2–6.7%, 8.4–8.6%, and 5.9–8.8% for K0, K1, and K2 application, whereas seed protein content increased by 3.7–8.0%, 1.6–6.4%, and 2.7–5.1% for K0, K1, and K2 application, respectively. Compared to K0, an increase in oil content in well-watered (3.8–5.2%) and drought stress (2.8–3.2%) conditions was observed after soil was applied with K fertilizer. In contrast, seed protein content decreased as the K application rate increased in both water regimes, with an average decline of 2.0–3.7% in well-watered and 4.1–5.2% in drought stress conditions, as contrasted with K0 plants (Figure 2C,D). The patterns were consistent across both experimental years. Two-way ANOVA showed that drought stress (DS) and K fertilizer rate had significant effects on seed K content (*p* < 0.05), while it was not affected by the interaction of DS and K rate.

**Figure 3:**
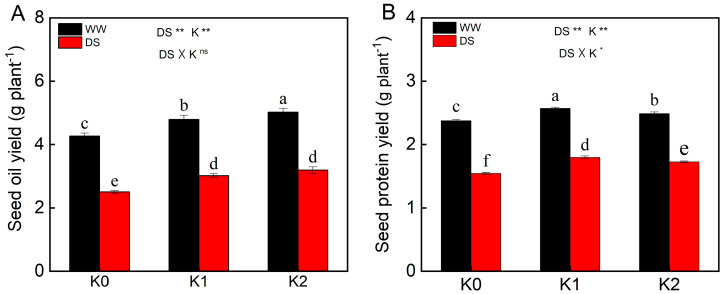
Effects of K application on seed oil (**A**) and protein (**B**) yield under different water treatments. K0, K1, and K2 were designated for 0, 60, and 120 kg K_2_O ha^−1^, respectively. WW—well-watered; DS—drought stress. Data are expressed as the means of three replications (mean ± SE, n = 3). Different lowercase letters show significant differences at *p* < 0.05 level. * and ** indicates significant difference at *p* < 0.05 and *p* < 0.01 probability level, respectively. ns means not significant.

DS significantly reduced seed oil (Figure 3A) and protein (Figure 3B) yield across all K treatments, while K application significantly increased oil and protein yield under both WW and DS conditions (*p* < 0.05). The interaction effect of DS × K on seed protein yield was significant (*p* < 0.05), with K application partially offsetting the yield loss caused by DS (Figure 3).

### 2.3. Changes in Sesamin and Sesamolin Content

**Figure 4:**
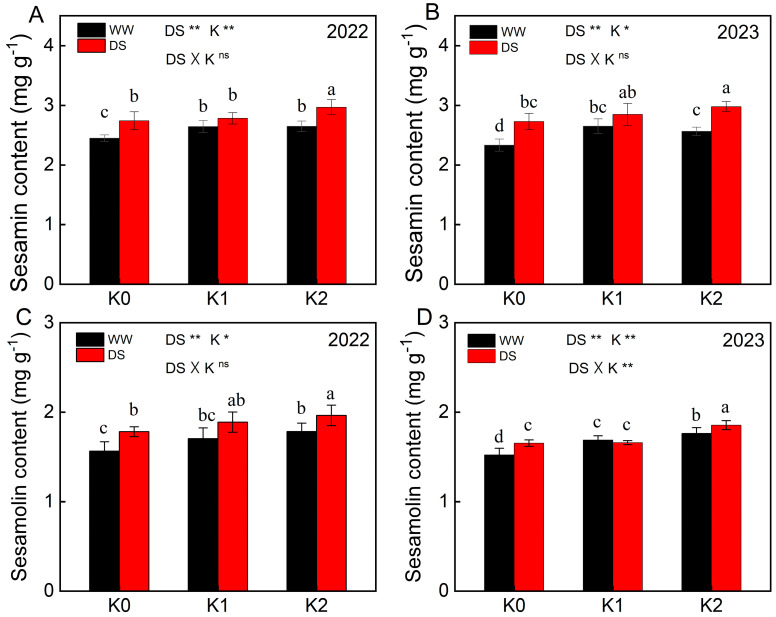
Effects of K application on seed sesamin (**A**,**B**) and sesamolin (**C**,**D**) content under different water treatments. K0, K1, and K2 were designated for 0, 60, and 120 kg K_2_O ha^−1^, respectively. WW—well-watered; DS—drought stress. Data are expressed as the means of three replications (mean ± SE, n = 3). Different lowercase letters show significant differences at *p* < 0.05 level. * and ** indicates significant difference at *p* < 0.05 and *p* < 0.01 probability level, respectively. ns means not significant.

As shown in Figure 4, potassium application led to a notable increase in both concentrations of sesamin and sesamolin under different water regimes, with a synergistic effect observed with the addition of K fertilizer. Specifically, under DS conditions, sesamin levels increased by 13.7% and 23.2% in treatments with K1 and K2 as compared to K0 (Figure 4A,B), while sesamolin levels increased by 11.2% and 22.4%, respectively (Figure 4C,D). Similarly, under DS conditions, sesamin content in seeds increased by 14.7% and 26.0% in K1 and K2 treatments compared to K0, and sesamolin content increased by 13.3% and 31.5%, respectively. Sesamin content in the embryo was not affected by the interaction effect of DS × K rate (*p* > 0.05) over two years.

### 2.4. Changes in Amino Acid Content

DS had a significant effect on the composition and content of seed amino acids (*p* < 0.05), while K application had no significant effect on total essential amino acids (EAAs) and total non-essential amino acids (NAAs) (*p* > 0.05). The main effect of DS was to significantly increase the content of 15 amino acids (*p* < 0.05), with no significant change in glycine content (*p* > 0.05) and a significant decrease in cysteine content (32.4%, *p* < 0.05) (Table 1). Under drought, the seed NAA and EAA content significantly increased (by 8.5% and 12.3%) compared with well-watered conditions. However, there was no difference in EAAs/NAAs. The main effect of K on amino acid content was negligible under DS, with only threonine, valine, isoleucine, and glutamic acid content significantly increasing following K application (*p* < 0.05). Compared with K0, K1 and K2 treatments increased total EAA by 1.4% and 0.9%, and total NAA by 0.5% and −0.4%, respectively (*p* > 0.05). Notably, K application significantly increased the proline (Pro) and tryptophan (Try) content under DS (*p* < 0.05), with K2 treatment increasing Pro and Try content by an average of 15.2% and 12.8% compared with K0 across two years. The amino acid composition and content were consistent between the two years (*p* > 0.05).

### 2.5. Changes in Fatty Acid Content and Composition of Sesame Seeds

The main effect of DS was to increase the oleic acid (C18:1), linolenic acid (C18:3) and arachidic acid (C20:0) content by an average of 9.5%, 7.2% and 11.4%, respectively, and decrease the palmitic acid (C16:0) and stearic acid (C18:0) content by an average of 11.4% and 12.0%, respectively (*p* < 0.05) (Table 2). K application further decreased palmitic acid under DS but had no effect under well-watered conditions. As compared to K0, the palmitic content decreased by 5.3% and 12.8% in K1 and K2 under drought stress, respectively. Concurrently, K significantly decreased the content of oleic and linolenic acids. Compared with K0, the oleic acid content was reduced by 4.6% and 6.4% in K1 and K2 under drought stress, whereas linolenic acid content declined by 5.6% and 12.9% in K1 and K2, respectively. Conversely, the linoleic acid content was increased by 5.5% and 9.3% in K1 and K2 under DS.

**Figure 5:**
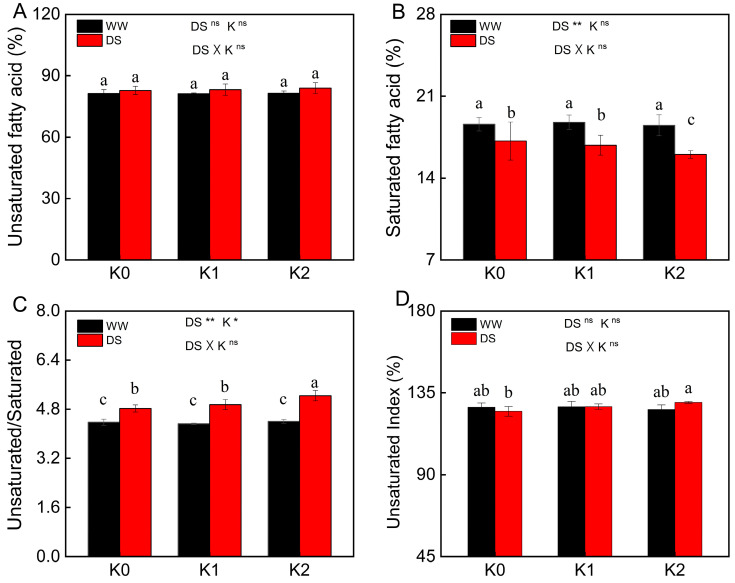
Effects of K application on seed fatty acid compositions unsaturated fatty acid (**A**), saturated fatty acid (**B**), unsaturated/saturated (**C**), and unsaturated index (**D**) under different water treatments. K0, K1, and K2 were designated for 0, 60, and 120 kg K_2_O ha^−1^, respectively. WW—well-watered; DS—drought stress. Data are expressed as the means of three replications (mean ± SE, n = 3). Different lowercase letters show significant differences at *p* < 0.05 level. * and ** indicates significant difference at *p* < 0.05 and *p* < 0.01 probability level, respectively. ns means not significant.

As a result, in Figure 5A, the unsaturated fatty acid (USFA) was not affected by DS, K rate, and their interactions. Compared with K0, the total saturated fatty acid (SFA) content decreased by 2.1% and 6.7% in K1 and K2 under drought stress, respectively (Figure 5B). Compared with WW conditions, DS induced a decline of 10.5% in SFA and an increment of 13.6% in unsaturated fatty acid (UFA)/SFA (Figure 5C). Specifically, compared to K0, the levels of UFA/SFA increased by 2.6% and 8.6% in K1 and K2 treatments under DS, respectively. The unsaturation index of fatty acids reflects the average number of carbon-carbon double bonds per fatty acid molecule in a sample. Drought stress resulted in an increment of 0.4% in the unsaturation index (UI) of the sesame seeds (Figure 5D). Exogenous potassium further increased the UI by 2.1% and 3.9% as compared to K0.

### 2.6. Correlation of Quality Traits in Sesame Seeds

**Figure 6:**
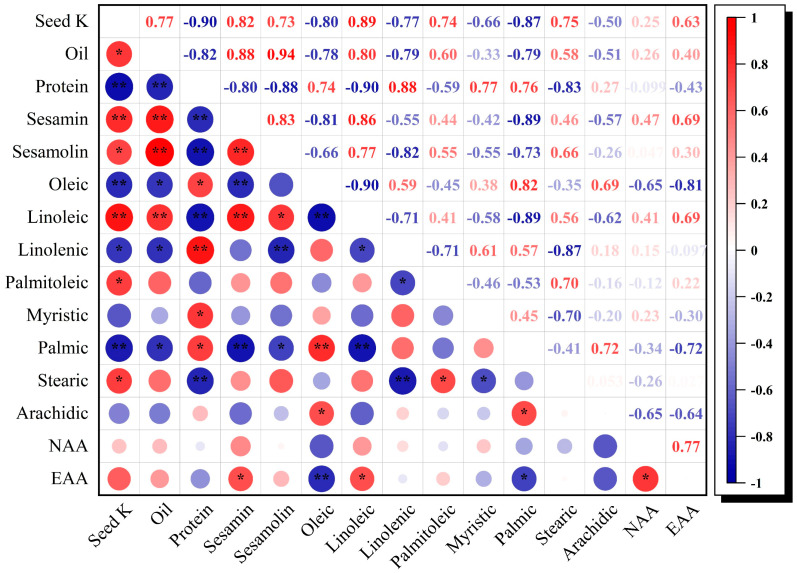
Correlation coefficients among seed K, oil, protein, amino acid, lignans and fatty acid compositions for K application under drought. n = 9, R_0.05_ = 0.666, R_0.01_ = 0.798. * and ** indicate significant differences at *p* < 0.05 and *p* < 0.01 probability level, respectively. A larger circle indicates a stronger correlation among the indicators.

The positive correlations were verified between seed K and seed oil content, as well as the stearic, palmitoleic, linoleic, sesamin, and sesamolin content (Figure 6). Contrarily, the protein, oleic, linolenic and palmitic of sesame seeds were negatively correlated with seed K. Furthermore, the sesamolin exhibited a strong correlation with polyunsaturated fatty acids (PUFAs), while it was not correlated with monounsaturated fatty acid (MUFA) content. The NAA was not correlated with fatty acid component indicators (except for seed oleic), seed K, oil or protein but was positively correlated with EAA.

### 2.7. Principal Component Analysis (PCA) of Quality Traits

**Figure 7:**
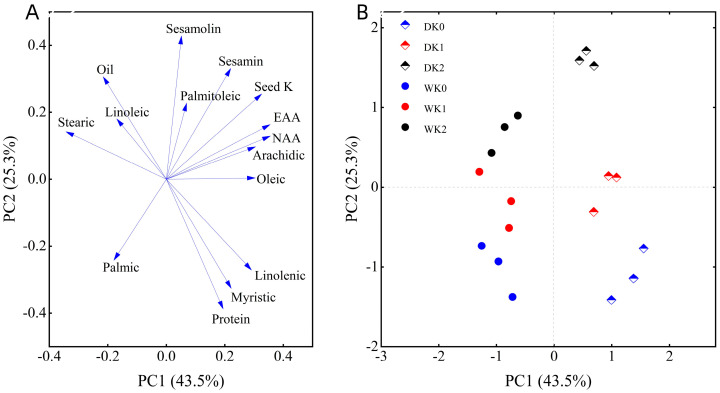
Principal component analysis (PCA) of oil biochemical traits for K application under different water treatments. (**A**) Loading plot and (**B**) Score plot. DK0, DK1, and DK2 were designated for 0, 60, and 120 kg K_2_O ha^−1^ respectively under drought conditions, while WK0, WK1, and WK2 were designated for 0, 60, and 120 kg K_2_O ha^−1^ respectively under well-watered conditions.

To reduce the dimensionality of fifteen seed quality indexes, PCA was applied, and two principal components were extracted based on the eigenvalue (EV > 1). According to the obtained results, the two main components (PC1 and PC2) exhibited 42.5% and 25.3% of the total variance, respectively, representing most of the information for those fifteen indicators (Figure 7A). As can be seen from Figure 7B, the samples from the drought stress and well-watered conditions were separated into four different regions. The samples of K2 treatment under DS appear in the right upper quadrant for PC1 and PC2. The indicators of seed K, oleic acid, sesamin, sesamolin, arachidic acid, palmitoleic acid, EAA, and NAA describe these samples. Concurrently, K2 application under WW appeared in the positive region for PC2 and the negative region for PC1. These samples are highly associated with seed oil, linoleic, and stearic acids. The K0 treatment under WW was characterized only by seed palmitic acid and is exhibited in the left lower quadrant for PC1 and PC2. Finally, the K0 treatment under DS was described by seed protein, linolenic and myristic acids, and occupied the positive region for PC1, while in the negative region for PC2.

## 3. Discussion

Sesame, as an oilseed crop, has a high demand for K during seed development, and adequate K supply is essential for oil synthesis and stress resistance [31]. Under drought stress (DS), plants increase K uptake and accumulation to maintain cell turgor and osmotic balance, which is an important adaptive mechanism to drought [32]. This study found that drought stress significantly increased seed K content by an average of 12.8% across all K treatments, which is consistent with the results of Fang et al. [8] in sesame. Exogenous K application further increased seed K content in a dose-dependent manner under DS and well-watered (WW) conditions (Figure 1A,B), with K2 treatment showing the strongest effect. The increased seed K accumulation under K application is due to the improved K uptake and transport capacity of sesame roots under drought stress [33], which provides a material basis for the regulation of seed quality formation.

### 3.1. Effects of Drought and K Application on Seed K, Oil and Protein

Drought decreased seed oil content and increased protein content (Figure 2), which is consistent with findings of Qureshi et al. [14]. This phenomenon is mainly due to the competition for carbon and nitrogen sources between oil and protein synthesis during seed maturation [34]. Under drought stress, sesame plants reduce the synthesis of storage lipids and increase the synthesis of storage proteins to improve stress resistance, as proteins can act as nitrogen reserves and osmo-protectants [35]. K application significantly increased seed oil content (Figure 2A,B) and decreased protein (Figure 2C,D) content under drought conditions, with a significant negative correlation between oil and protein content (Figure 6). This is because K acts as a cofactor for key enzymes in oil synthesis [31], and promotes the conversion of carbon skeletons to oil by regulating photosynthetic metabolism and sucrose transport [27]. The negative correlation between oil and protein content is also attributed to the allelic regulation of their synthesis genes, which is a common phenomenon in oilseed crops such as cotton [36] and rape [27]. The consistent results between the two years confirm the stability and reliability of K application in ameliorating drought stress damage in sesame.

### 3.2. Regulation of K on Lignans and Amino Acids Under Drought

Drought stress significantly increased the content of sesame seed lignans (sesamin and sesamolin), which is consistent with the results of Kermani et al. [17]. Lignans are important secondary metabolites in sesame, which can scavenge ROS and mitigate oxidative damage under stress [37,38], and their accumulation is an important adaptive mechanism of sesame to drought stress. K application further increased lignan content under DS, with a higher increment than under WW, indicating that K has a synergistic effect with drought stress on lignan accumulation (Figure 4A–D). The synthesis of lignans is closely related to amino acid metabolism, especially tryptophan (Try) and proline (Pro). Try is a precursor of the phenylpropanoid pathway, which is converted into coniferyl alcohol and then forms lignans through stereospecific dimerization [38]. Pro is an important osmo-protectant, which can improve plant drought tolerance by maintaining cell osmotic balance and scavenging ROS [16]. This study found that K application did not affect total essential amino acid and non-essential amino acid content under DS (Table 2), but significantly increased Pro and Try contents, which is the main reason for the promotion of lignan accumulation by K application. In addition, K application reduces ROS production in sesame plants under drought stress [8], which further promotes the synthesis and accumulation of lignans, as ROS can inhibit the activity of key enzymes in the lignan synthesis pathway [38]. The significant positive correlation between seed K content and lignan content (Figure 6) further confirms the regulatory role of K in lignan accumulation.

### 3.3. Effects of K on Fatty Acid Composition and Oil Nutritional Quality

The quality of sesame oil is mainly determined by its fatty acid composition, especially the proportion of unsaturated fatty acids (UFAs) and the linoleic acid content [39]. Drought stress altered the fatty acid composition of sesame seeds by increasing oleic, linolenic, and arachidic acids and decreasing saturated fatty acids and linoleic acid, which is consistent with the results of Qureshi et al. [14] and Keshavarz [40]. This is mainly due to the inhibition of desaturase activity under drought stress, which reduces the conversion of oleic acid (C18:1) to linoleic acid (C18:2). Desaturase is a key enzyme in fatty acid unsaturation, and its activity is highly sensitive to drought stress and ROS [41]. In addition, oxidative degradation of polyunsaturated fatty acids under drought stress also leads to a decrease in linoleic acid content [41].

Potassium, which acts as an activator of Δ12-desaturase, can enhance the activity of desaturase under drought stress [42], promote the desaturation of saturated fatty acids to unsaturated fatty acids and the conversion of monounsaturated fatty acids (oleic acid) to polyunsaturated fatty acids (linoleic acid), thus increasing linoleic acid and decreasing oleic acid content. The significant negative correlation between oleic and linoleic acid (Figure 6) confirms this conversion relationship. K can reduce the oxidative degradation of polyunsaturated fatty acids under drought stress by improving the antioxidant capacity of sesame seeds (increasing lignan content), thus maintaining the stability of fatty acid composition. Linolenic acid (C18:3) is a highly unsaturated fatty acid that is easily oxidized under stress [41]. K application decreases linolenic acid content to reduce oxidative damage to oil, which is an important adaptive mechanism to improve oil quality under drought stress. Thus, there was a strong positive correlation between oil and linoleic acid (Figure 6).

Concurrently, K activates fatty acid elongase [43], which promotes the elongation of short-chain saturated fatty acids (SFAs) to long-chain SFAs, thus increasing the stearic acid content and decreasing the palmitic acid content. Palmitic acid has a negative intrinsic correlation with oil content [44], and K-induced oil accumulation inevitably leads to a decrease in palmitic acid content (a 1% increase in palmitic acid leads to a 1.4% decrease in oil content). K is an activator of many enzymes involved in converting fatty acids from saturated to unsaturated forms [43]. The connection between distinct enzymatic processes of fatty acid production is the primary determinant of the seed fatty acid characteristics [45].

### 3.4. Comprehensive Evaluation and Practical Implications

PCA is an effective method for the comprehensive evaluation of crop quality traits [46]. The samples of different treatments were clearly separated, indicating that water regime and K application have significant and characteristic effects on sesame seed quality (Figure 7). DK2 (DS + K2) samples were distributed in the upper-right quadrant of PC1-PC2, and were characterized by high seed K, lignans, linoleic acid, UFA/SFA and UI, which are the key quality traits for food and pharmaceutical use (Figure 7A). This indicates that the K2 application under drought can comprehensively improve sesame seed quality, which is consistent with the correlation analysis results. The traits with high positive loadings on PC2 (seed K, oil, linoleic acid, lignans) are all the core quality traits of sesame, and their loadings are positively correlated with K application rate, further confirming the core regulatory role of seed K in sesame seed quality formation under drought stress (Figure 7B). The PCA results provide a comprehensive evaluation method for sesame seed quality under drought stress and K application, and can be used for the screening of optimal agronomic measures. From the perspective of field agronomic production, drought is a major limiting factor for sesame yield and quality in rain-fed dryland areas across southern and northern China. Traditional fertilization management usually ignores potassium supplementation, which further aggravates yield loss and quality decline of sesame under water deficit. This study verifies that rational potassium fertilization is a low-cost, easy-to-operate agronomic regulation measure for drought resistance and quality improvement of sesame. The optimal rate of 120 kg K_2_O ha^−1^ proposed in this study is suitable for popularization and application in drought-prone sesame planting areas, which can effectively balance yield and nutritional quality of sesame seeds, and offers economic benefits for local oil crop production. On the basis of this study, subsequent experiments will combine transcriptome and metabolome technologies to explore the molecular regulatory network of potassium fertilization on the expression of fatty acid desaturase and lignan synthesis-related genes in sesame under drought stress. Meanwhile, multi-site field verification and genotype screening will be carried out to further optimize the potassium application scheme for different sesame varieties and different drought degrees.

## 4. Materials and Methods

### 4.1. Experimental Design

A two-factor randomized complete block design was conducted for two consecutive years (2022–2023) in a polythene shelter with a switchable roof at the Jiangxi Agricultural University Science and Technology Experimental Station (28°76′ N, 115°84′ E) in Nanchang, China. The two experimental factors were: (1) water regime: well-watered (WW, 75 ± 5% soil relative water content) and drought-stressed (DS, 50 ± 5% soil relative water content); (2) potassium fertilizer rate: K0 (0 kg K_2_O ha^−1^, 0 g pot^−1^), K1 (60 kg K_2_O ha^−1^, 0.75 g pot^−1^), K2 (120 kg K_2_O ha^−1^, 1.5 g pot^−1^). Each treatment was set up with 3 biological replicates, and replication contained five pots. All index determinations were repeated 3 times as technical replicates to ensure data reliability.

Each pot was full of 14.5 kg air-dried red soil derived from the topsoil layer of the experimental location, with each pot considered a replicate. The soil is quaternary red clay, with clay-to-loam texture, high kaolinite clay content, and less K-containing minerals, which is a typical Plinthosol, based on IUSS guidelines [47]. Fresh soil was used each year to avoid residual effects and ensure independent experiments. The basic soil properties at a depth of 0–20 cm was shown in Table 3.

The K fertilizer (potassium sulphate, 50% K_2_O) was divided into two splits that 50% as a basal dose and the remaining 50% at the flowering initiation period. For basal application, the required amount of K_2_SO_4_ was thoroughly mixed with the soil to ensure uniform distribution. For the topdressing at flowering, the fertilizer was dissolved in a small volume of water, evenly sprinkled onto the soil surface, and then immediately mixed into the top 5–8 cm of soil followed by light irrigation to incorporate it into the root zone. In addition, phosphorus (superphosphate, 12% of P_2_O_5_) and nitrogen (urea, 46% of N) fertilizers were applied at rates of 90 kg P_2_O_5_ ha^−1^ and 120 kg N ha^−1^ before sowing, respectively. There were 90 pots and each pot was sown with 3 seeds and thinned to a single plant per pot after emergence. Adequate water was supplied to all pots until flowering. At the initial flowering stage (26 July 2022, and 21 July 2023), watering was withheld for half of the pots to induce drought stress. The drought treatment was maintained for six days, during which the soil moisture was kept at 50 ± 5% of the soil relative water content (SRWC). The remaining half continued to be watered, maintaining a soil moisture level of 75 ± 5% SRWC as the control group. During the drought stress treatment, soil samples (0–20 cm) were collected daily from different pots at 6:30 p.m. local time using a hole drill, following the method described by Liu et al. [48]. Soil water content was expressed as g water g^−1^ dried soil. Meanwhile, the sesame plants in the control half were continuously watered to maintain 75 ± 5% soil relative water content in the early morning.

### 4.2. Sample and Processing

Sesame plants in each plot were harvested at maturity (90 days after germination) to estimate seed yield. The harvested seeds were dried at 103 ± 2 °C for 2 h using a ventilated oven and then dried at 60 °C for 24 h to a constant weight. The seeds were ground to a fine particle size, screened through a 1 mm sieve and partially digested using a mixture of concentrated H_2_SO_4_-H_2_O_2_ to determine the K concentrations using a flame spectrophotometer (Shanghai, FP6410, China) according to Hu et al. [49].

### 4.3. Determination of Amino Acid Content

First, 0.1 g seeds were weighed and extracted with 10 mL of 0.1% phenol and 6 mol L^−1^ HCl. The mixture was then ground into a slurry and hydrolyzed in an oven at 110 °C for 20 h. After cooling, 1 mL of the hydrolysate was taken, dried, and dissolved in 1 mL of 0.1 mol L^−1^ hydrochloric acid aqueous solution, and then filtered through a membrane for derivatization. The amino acid standard mixture was prepared by mixing 17 different amino acids in 0.1 mol L^−1^ HCL, and components were determined by using a high-performance liquid chromatography instrument (Agilent 1260, MA, USA) according to Liu et al. [50].

### 4.4. Determination of Crude Protein and Crude Fat

Next, 0.2 g seeds were weighed and digested with H_2_SO_4_-H_2_O_2_ for protein determination using Kjeldahl nitrogen analyzer (Kjeltec 8400, Hillerød, Denmark) to obtain the total nitrogen (N) content. The formula for calculating crude protein content is as follows:

(1) Crude protein content (g kg^−1^) = total nitrogen (N) × 6.25.

Concurrently, seed crude fat contents were extracted using a soxhlet extraction kit (KIMBLE®, DWK Life Sciences, Millville, NJ, USA) [51].

Specifically, 3 g (W1) of sesame seed was placed in a filter paper cylinder, with a small amount of absorbent cotton covered on the top to prevent sample splashing and loss during reflux, and weighed (W2) before being put into a Soxhlet extraction tube. Approximately 1/2 of the volume of petroleum ether was injected into the extraction bottle, the condenser tube inlet pipe was opened to start the 60 °C water bath heating extraction to maintain a stable reflux rate of 6–10 cycles per hour, and the extraction time was 8–12 h until the eluted petroleum ether solution became colorless, indicating the complete extraction of crude lipids from sesame samples. The sample was dried and weighed (W3), after which the oil content was calculated:

(2) Oil content (%) = (W2 − W3)/W1 × 100%

### 4.5. Determination of Fatty Acid

The fatty acid composition was measured with slight modifications [36]. Then, 0.2 g of sesame seeds was weighed and placed into a 10 mL test tube. Then, a 1 mL mixture of petroleum ether and diethyl ether was added, followed by 1 mL of potassium hydroxide and methanol solution. After vortex oscillation and reaction for 1 h, the extraction was centrifuged at 4500× *g* for 2 min and analyze by using gas chromatography–mass spectrometry (Agilent 7000E, Santa Clara, CA, USA). Samples were quantified by the external standard method, and the external standard was a mixture of 37 fatty acid methyl ester standards (ANPEL Laboratory Technologies, Inc., Shanghai, China), which was used to calibrate and quantify fatty acids.

### 4.6. Determination of Sesamin and Sesamolin Content

We ground 0.1 g of seeds into a slurry with 1.0 mL of methanol at 4 °C for 1 h. The samples were then centrifuged at 2000× *g* for 3 min at 25 °C, and the supernatant was filtered through a 0.45 µm PFTE filter before HPLC (Agilent 1260, MA, USA) analysis [17].

### 4.7. Data Analysis

Data analysis was performed using SPSS 22.0 (IBM, Armonk, NY, USA) and Microsoft Excel 2023. Since some results were inconsistent across years and the initial combined data showed interactions with year, the results were separately analyzed for each year. The two-way analysis of variance (ANOVA) was performed for multiple comparisons of means among different treatments at the *p* < 0.05 significance level, where cultivar and K treatment were fixed effects. Correlation analysis was performed using the Pearson correlation coefficient method, and principal component analysis (PCA) was performed using the standardized data of seed quality traits. Graphs were plotted using Origin Pro 2021 (OriginLab, Northampton, MA, USA), with all data presented as mean ± standard error (SE) (n = 3).

## 5. Conclusions

Drought stress significantly altered the chemical composition of sesame seeds, reducing seed oil content but increasing protein content. External K ameliorated the adverse effects of drought on seed oil characteristics and fatty acid composition by enhancing seed K content, which improves seed oil nutritional quality of sesame. Specifically, drought stress significantly increased the content of oleic and linolenic acids but decreased the stearic, palmitic, and linoleic acid content, which consequently decreased saturated fatty acids. Concurrently, under drought stress, K application inversely increased the percentage of linoleic acid and reduced oleic and linolenic acid. Therefore, the unsaturated index and UFA/SFA were increased along with increasing K application rates. According to the correlation results, we could suggest that seed K acts as a critical factor for oil, protein, and fatty acids profile under drought stress. Finally, 120 kg K_2_O ha^−1^ is recommended as the practical soil-applied K rate to improve sesame seed quality under drought stress.

## Figures and Tables

**Table 1:** Effect of K on seed amino acid components under different water regimes (mg g^−1^).

Amino Acids	Well-Watered (WW)			Drought Stress (DS)		
	K0	K1	K2	K0	K1	K2
Essential amino acids (NAA mg g^−1^)						
Lys	6.28 b	6.24 b	6.59 b	7.14 a	7.21 a	7.42 a
Trp	11.32 b	11.04 b	11.74 b	13.24 a	13.50 a	13.27 a
Phe	9.16 b	9.29 b	9.80 a	10.15 a	10.25 a	10.06 a
Met	4.21 c	4.16 c	4.14 c	4.57 b	4.79 ab	4.88 a
Thr	7.31 bc	7.21 c	7.63 b	8.60 a	8.73 a	8.68 a
Ile	7.28 d	7.43 d	7.78 c	8.08 b	8.20 ab	8.44 a
Leu	14.67 b	14.72 b	15.24 ab	15.82 a	16.00 a	15.85 a
Val	7.68 d	7.94 d	8.29 c	9.34 a	9.32 a	9.04 b
Total EAA	67.90 c	68.04 c	71.20 b	76.95 a	78.01 a	77.64 a
Non-essential amino acids (NAA mg g^−1^)						
Asp	19.73 b	19.95 b	20.39 b	23.49 a	23.94 a	23.60 a
Glu	41.73 c	43.21 bc	43.15 bc	46.43 a	45.53 a	43.83 b
Gly	11.88 a	12.11 a	12.31 a	11.89 a	12.11 a	12.18 a
His	5.18 b	5.26 b	5.18 b	6.11 a	6.02 a	6.14 a
Arg	30.20 b	30.14 b	31.19 ab	32.27 a	32.63 a	32.85 a
Ala	10.31 b	10.23 b	10.79 ab	11.56 a	11.75 a	11.61 a
Pro	9.69 b	9.68 b	9.80 b	10.38 ab	10.48 ab	10.89 a
Tyr	8.04 b	7.92 b	8.11 b	8.61 ab	9.06 a	9.02 a
Cys	0.12 a	0.12 a	0.13 a	0.08 b	0.08 b	0.09 b
Total NAA	136.88 b	138.62 b	141.78 b	150.83 a	151.60 a	150.22 a
EAA/NAA	0.50 ab	0.49 b	0.50 ab	0.51 ab	0.52 a	0.52 a

K0, K1 and K2 represent 0, 60 and 120 kg K_2_O ha^−1^ respectively, WW—well-watered; DS—drought stress. Data are expressed as the means of three replications (mean ± SE, n = 3). Different lowercase letters show significant differences at *p* < 0.05 level.

**Table 2:** Effect of K on fatty acid components under different water regimes (g 100 g Oil^−1^).

Treatments		Myristic C14:0	Palmitic C16:0	Stearic C18:0	Arachidic C20:0	Palmitoleic C16:1	Oleic C18:1	Linoleic C18:2	Linolenic C18:3
WW	K0	0.24 b	12.60 a	5.08 ab	0.69 b	0.13 a	35.76 bc	45.13 a	0.37 c
	K1	0.23 b	12.70 a	5.12 a	0.71 ab	0.13 a	35.21 c	45.51 a	0.37 c
	K2	0.23 b	12.40 a	5.18 a	0.71 ab	0.13 a	37.25 abc	43.74 ab	0.37 c
DS	K0	0.26 a	11.91 a	4.22 d	0.79 a	0.13 a	40.99 a	41.29 b	0.42 a
	K1	0.24 b	11.27 ab	4.52 c	0.78 a	0.13 a	39.10 ab	43.56 ab	0.40 b
	K2	0.23 b	10.21 b	4.80 bc	0.78 a	0.14 a	38.35 abc	45.12 a	0.37 c

K0, K1 and K2 represent 0, 60 and 120 kg K_2_O ha^−1^ respectively, WW—well-watered; DS—drought stress. Data are expressed as the means of three replications (mean ± SE, n = 3). Different lowercase letters show significant differences at *p* < 0.05 level.

**Table 3:** Chemical properties of the soil used for the experiments in 2022 and 2023.

Years	pH	Organic Matter (g kg^−1^)	Total N (g kg^−1^)	Alkali Hydrolysable N (mg kg^−1^)	Available P (mg kg^−1^)	Available K (mg kg^−1^)
2022	5.7	12.8	0.8	37.7	22.6	95.3
2023	5.6	13.5	0.9	31.2	19.6	89.3

## Data Availability

The raw data supporting the conclusions of this article will be made available by the authors on request. The data are not publicly available due to privacy and ethical restrictions.

## Abbreviations

The following abbreviations are used in this manuscript:

WW	Well-watered
DS	Drought stress
K	Potassium
UFA	Unsaturated fatty acids
SFA	Saturated fatty acids
MUFA	Monounsaturated fatty acid
PUFA	Polyunsaturated fatty acids
UI	Unsaturation index
PCA	Principal component analysis
ROS	Reactive oxygen species
